# A Study of the Geo-Herbalism of Evodiae Fructus Based on a Flow-Injection Mass Spectrometric Fingerprinting Method Combined with Chemometrics

**DOI:** 10.3390/molecules20022658

**Published:** 2015-02-03

**Authors:** Yang Zhao, Xin Zhou, Yun-Ling Zhao, Xiao-Jian Gong, Chao Zhao

**Affiliations:** 1 Key Laboratory for Information System of Mountainous Areas and Protection of Ecological Environment, Guizhou Normal University, Guiyang 550001, China; E-Mails: zhaoyang5620328@163.com (Y.Z.); zzzhaoyunling@163.com (Y.-L.Z.); gongxiaojian1@163.com (X.-J.G.); chaozhao@126.com (C.Z.); 2 The Research Center for Quality Control of Natural Medicine, Guizhou Normal University, Guiyang 550001, China

**Keywords:** geo-herbalism, Evodiae Fructus, flow-injection mass spectrometric fingerprinting method, chemometrics

## Abstract

A flow-injection mass spectrometric (FIMS) fingerprinting method in combination with principal component analysis (PCA) was used to study the geo-herbalism of Evodiae Fructus (EF) samples. Twenty four EF samples from different regions in China were collected and analyzed. The PCA scores plot showed that the samples from Guizhou Province were scattered in different groups, however, most of the samples from other provinces were basically scattered in the same group. Nine characteristic compounds responsible for the classification of the samples were tentatively characterized. These nine compounds might help differentiating EF samples from different regions.

## 1. Introduction

Evodiae Fructus (EF), the dried, nearly ripe fruits of *Evodia rutaecarpa* (Juss.) Benth., *Evodia rutaecarpa* (Juss.) Benth. var. *officinalis* (Dode) Huang or *Evodia rutaecarpa* (Juss.) Benth. var. *bodinieri* (Dode) Huang, is listed in the China Pharmacopoeia (2010 edition). The herb is reported to have anti-inflammatory [1,2], anti-nociceptive [3], anti-microbial [4], anthelmintic [5], thermo-regulatory [6], anti-hypertensive [7,8], anti-anoxic [9,10] and anti-diarrheal [11] properties.

In herb studies, there is no doubt that their curative effects have a close relationship with their medicinal components. The famous ancient medicinal physicians in China, such as Hongjing Tao in the Liang Dynasty, Jiamo Chen in the Ming Dynasty and Simiao Sun in the Tang Dynasty, and others attached great importance to herb resources. Currently, we still place great emphasis on geo-herbalism of an herb. Geo-herbalism refers to a certain biological variety in the specific environment. A geo-authentic herb is proven to be of good quality, high clinical curative effect, and is traditionally recognized as a regional characterized herb [12]. EF is regarded as a geo-authentic herb in Shanxi and Shandong provinces in early years, but now it is well known as one of the geo-authentic herbs in Guizhou province and is widely planted by Good Agricultural Practices (GAP) planting bases. Previous quality assessment studies on EF mainly focused on the quantification of chemical compounds [13] and its fingerprinting method [14]. However, few studies have been conducted to verify its geo-herbalism.

Flow injection mass spectrometric (FIMS) fingerprinting method combined with chemometrics has been widely applied for differentiation plants between different species and for quality assessment on TCM or TCM-derived dietary supplements [15,16,17]. The approach has been proven to be fast, useful, easy and efficient in authentication and classification of complex systems and related products.

The aim of the present study was to explore the geo-herbalism of EF from Guizhou and other provinces based on FIMS combined with chemometrics.

## 2. Results and Discussion

### 2.1. PCA of Mass Spectrometric Fingerprints

PCA involves a mathematical procedure for reducing the dimensionality of multidimensional matrices while retaining a large amount of the original information in the data set. Generally, it is easy to visually compare the mass spectra of a few samples, but the comparison of hundreds of samples quickly overwhelms our ability to see patterns. The advantage of using PCA to process the MS fingerprints is automation and simplification. PCA provides visual patterns that everyone can easily understand and this also avoids subjective decisions.

**Figure 1:**
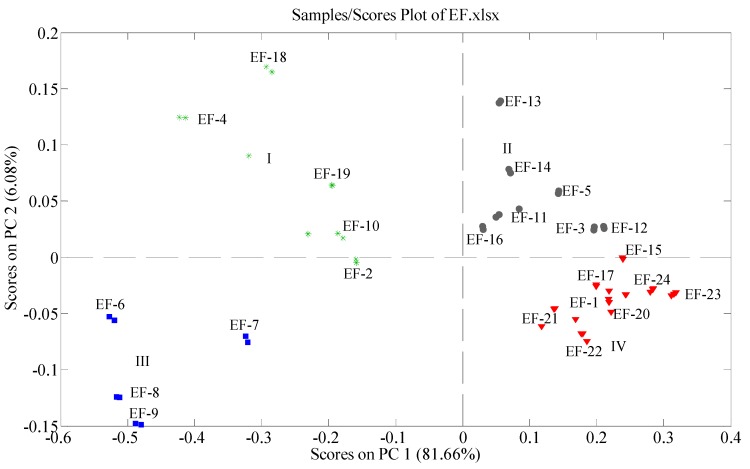
PCA scores plot of the tested EF samples obtained in positive ion mode. Each mark represents an analysis of a sample, and the labels represent corresponding samples listed in Table 1.

In the present study, the scores plot of PC1 *versus* PC2 was examined relating to different original samples. The scores plot is shown in Figure 1, which shows a very interesting grouping phenomenon. All 24 EF samples tested in the present study are classified in four groups, I, II, III and IV. Samples 2, 4, 10, 18 and 19 which get PC1 scores below 0 and PC2 scores above 0 (except sample 2 that gets a PC2 score below 0) are clustered in group I. Samples 3, 5, 11, 12, 13, 14 and 16 which gets both PC1 and PC2 scores above 0 are clustered in group II. Samples 6, 7, 8 and 9 which get both PC1 and PC2 scores below 0 are clustered in group III. Samples 1, 15, 17, 20, 21, 22, 23 and 24 which get PC1 scores above 0 and PC2 scores below 0 are clustered in group IV. From the results and the source information of the samples, we can see that the samples from Guizhou Province are widely scattered (in Groups I, II and III) in the scores plot, however, the samples from the other provinces are basically scattered in group IV (except sample 19 in group I). This demonstrates that: (1) the chemical profiles of the samples from Guizhou and other provinces are different; (2) in Guizhou Province, the samples from different districts also show different chemical profiles. It is worth noting that sample 19 was purchased in a pharmacy in Zhongkang in Hunan Province, but possibly was imported from Guizhou Province.

**Table 1:** Source information of EF samples tested in the present study.

Sample ID	Source	Sample ID	Source
EF-1	Guanling, Tongren, Guizhou	EF-13	Guiyang Pharmacy, Guizhou
EF-2	Shiqian, Tongren, Guizhou	EF-14	Qianxinan, Guizhou
EF-3	Jiangkou, Tongren, Guizhou	EF-15	Liupanshui, Guizhou
EF-4	Jiangkou, Tongren, Guizhou	EF-16	Guiyang, Guizhou
EF-5	Jiangkou, Tongren, Guizhou	EF-17	Zunyi, Guizhou
EF-6	Dejiang, Tongren, Guizhou	EF-18	Yuping, Tongren, Guizhou
EF-7	Dejiang, Tongren, Guizhou	EF-19	Hunan
EF-8	Dejiang, Tongren, Guizhou	EF-20	Yueyang, Hunan
EF-9	Dejiang, Tongren, Guizhou	EF-21	Xiangtan, Hunan
EF-10	Liupanshui, Guizhou	EF-22	Ankang, Shanxi
EF-11	Liupanshui, Guizhou	EF-23	Jinhua, Zhejiang
EF-12	Zunyi, Guizhou	EF-24	Taizhou, Zhejiang

Scores plots show the distribution of the samples intuitively, from which we can see the similarities of the objects of study. Further, it will be meaningful to pinpoint the compounds from the chemical profiles which cause the distribution of the samples. Generally, a loadings plot clarifies not only how much a particular variable contributes to the PC but also how well that PC takes that variable into account over the data points.

The ions at *m*/*z* 286, 288, 302, 304, 314, 340, 356 and 366 were found to be responsible for the separation of samples in the scores plot (data not shown). On the one hand, the samples with high intensity of the ion at *m*/*z* 302 would get high PC1 scores, whereas the samples with high intensities of the ions at *m*/*z* 286, 288, 314, 340 and 366 would get low PC1 scores. On the other hand, the samples with high intensities of the ions at *m*/*z* 286, 314 and 356 would obtain high PC2 scores, whereas, the ones with high intensities of the ions at *m*/*z* 304 and 340 would obtain low PC2 scores. Certainly, the position of each sample in PCA scores plot depends upon the combined effects of these ions.

EF-32 had the highest intensity of the ion at *m*/*z* 302, making it be located at the rightmost place in the scores plot. In contrast, EF-6 had the lowest intensity of the ion at *m*/*z* 302, making it be located at the leftmost place in the scores plot. Similarly, EF-9 had the highest intensities of the ions at *m*/*z* 304 and 340, which made it be positioned at the bottom in the scores plot, whereas, EF-18 had the lowest intensities of the ions at *m*/*z* 304 and 340, making it be positioned on the top in the scores plot.

### 2.2. Identification of the Chemical Markers

**Figure 2:**
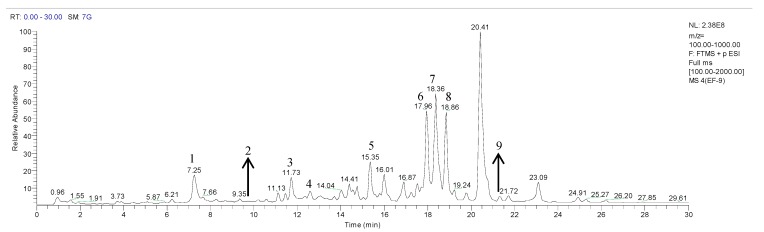
Representative total ion current chromatogram in positive ion mode.

Figure 2 shows the representative total ion current chromatogram obtained in positive ion mode, in which the characteristic peaks are marked from no. 1 to no. 9. The retention times (*t*_R_, min), [M+H]^+^ weights, [M+H]^+^ formulas, errors (ppm) between theoretical and measured [M+H]^+^ weight values, MS^2^ and MS^3^ ions of each characteristic peak as well as their assignment are summarized in Table 2.

The ion at *m*/*z* 302 was from peak 1 with a retention time at 7.3 min, of which the HRMS measurement was 302.1287 (C_19_H_16_ON_3_, −0.294 ppm). In the MS^2^ spectrum, the protonated ion at *m*/*z* 302 yielded a base peak at *m*/*z* 287, corresponding to the fragment ion losing a free radical CH_3_. This peak was characterized as dehydroevodiamine [18].

The ion at *m*/*z* 304 was from peak 2 with a retention time at 9.7 min, of which the HRMS measurement was 304.1080 (C_18_H_14_O_2_N_3_, −0.175 ppm). In the MS^2^ spectrum, the protonated ion at *m*/*z* 304 yielded a base peak at *m*/*z* 286 [M−H_2_O+H]^+^. The ion at *m*/*z* 288 [M−CH_4_+H]^+^ was observed as another dominant ion. This peak was characterized as evodiamine [14,18].

**Table 2:** Information of retention time (*t*_R_, min), [M+H]^+^ weight, [M+H]^+^ formula, error (ppm) between theoretical and measured values, MS^2^ and MS^3^ ions of the characteristic peaks.

Peak No.	t_R_ (min)	[M+H]^+^ Weight	[M+H]^+^ Formula	Error (ppm)	MS^2^ Ions	MS^3^ Ions	Identification
1	7.3	302.1287	C_19_H_16_ON_3_	−0.294	MS^2^ [302]: 302(56), 287(100), 286(19)	MS^3^ [302→287]: 286(100), 272(8)	Dehydroevodiamine
2	9.7	304.1080	C_18_H_14_O_2_N_3_	−0.175	MS^2^ [304]: 288(15), 286(100)		Evodiamine
3	11.7	288.1127	C_18_H_14_ON_3_	−1.522	MS^2^ [288]: 286(15), 284(23), 273(100), 271(86), 270(8), 261(6), 260(10), 245(7), 244(8), 243(15), 201(13), 199(12), 185(15), 169(6)		Rutaecarpine
4	12.6	356.2581	C_23_H_34_O_2_N	−0.858	MS^2^ [356]: 338(100)	MS^3^ [356→338]: 296(35), 284(12), 282(16), 270(13), 268(8), 256(10), 242(12), 240(15), 228(15), 226(26), 212(47), 200(13), 199(6), 198(12), 186(100), 184(11), 173(83)	1-Methyl-2-[7-hydroxy-(*E*)-9-tridecenyl]-4(1*H*)-quinolone
5	15.4	286.2163	C_19_H_28_ON	−0.842	MS^2^ [286]: 284(9), 201(9), 199(10), 186(16), 173(100), 160(8)		4-Hydroxy-1-methyl-2-nonyl-quinolinium
6	17.9	314.2474	C_21_H_32_ON	−1.404	MS^2^ [314]: 314(30), 313(9), 201(9), 199(8), 186(27), 185(14), 184(9), 173(100), 160(7)		Evodiaxinine
7	18.4	340.2632	C_23_H_34_ON	−0.856	MS^2^[340]: 341(79), 340(82), 298(21), 284(22), 270(14), 256(10), 242(11), 228(11), 200(17), 186(100), 173(56), 172(6), 160(6)		Evocarpine
8	18.8	366.2788	C_25_H_36_ON	−0.932	MS^2^ [366]: 340(12), 324(17), 310(13), 298(10), 296(11), 284(29), 282(12), 270(14), 268(6), 256(11), 254(6), 242(10), 240(11), 228(14), 226(13), 212(16), 201(17), 200(16), 199(18), 198(8), 186(100), 184(13), 173(70), 172(6)	MS^3^ [366→186]: 199(100), 196(20), 156(28), 142(23), 139(59), 133(24), 129(30), 121(26), 110(33), 98(33), 92(22), 90(21), 77(23), 71(46), 55(24), 53(23), 50(25)	1-Methyl-2-pentadecadienyl-4(1*H*)-quinolone
9	21.4	356.2943	C_24_H_38_ON	−1.379	MS^2^ [356]: 356(96), 284(7), 270(7), 256(6), 242(6), 200(6), 199(6), 186(56), 173(100), 160(8)	MS^3^ [356→173]: 352(11), 325(14), 323(10), 322(10), 317(13), 284(43), 274(23), 270(10), 256(10), 208(9), 206(10), 201(47), 199(37), 158(65), 155(18), 151(9), 144(22), 137(11), 135(9), 132(100), 128(10), 123(24), 122(10), 116(9), 115(9), 102(9), 100(17), 74(9), 64(22),	1-methyl-2-tetradecyl-4(1*H*)-quinolone

The ion at *m*/*z* 288 was from peak 3 that was eluted at a retention time of 11.7 min. The HRMS measurement of this peak was 288.1127 (C_18_H_14_ON_3_, −1.522 ppm). In the MS^2^ spectrum, the protonated ion at *m*/*z* 288 yielded a base peak at *m*/*z* 273 [M−CH_3_+H]^+^. This peak was characterized as rutaecarpine [14,18].

Two peaks (peak 4 and peak 9) with retention times at 12.6 and 21.4 min had ions at *m*/*z* 356. The HRMS measurement of the protonated [M+H]^+^ ion of peak 4 was 356.2581, suggesting the chemical composition C_23_H_34_O_2_N (−0.858 ppm). In the MS^2^ experiment, the ion at *m*/*z* 338 was found to be the base peak. It was tentatively characterized as 1-methyl-2-[7-hydroxy-(*E*)-9-tridecenyl]-4(1*H*)-quinolone according to the data in [19], which is the only publication reporting this compound to the best of our knowledge. HRMS measurement of peak 9 gave a [M+H]^+^ ion at *m*/*z* 356.2943, suggesting the chemical composition C_24_H_38_ON (−1.379 ppm).

The fragment ion at *m*/*z* 173 was found to be the base peak in MS^2^ experiment. This compound was tentatively identified as 1-methyl-2-tetradecyl-4(1*H*)-quinolone according to the MS behavior reported in [20].

The ion at *m*/*z* 286 was from peak 5, occurring at a retention time of 15.4 min. HRMS measurement of the peak gave a [M+H]^+^ ion at *m*/*z* 286.2163 (C_19_H_28_ON, −0.842 ppm). The protonated ion yielded a base peak at *m*/*z* 173 in the MS^2^ spectrum. Comparison of the MS behavior of peak 5 with that reported in the literature [14] indicated that it might be 4-hydroxy-1-methyl-2-nonylquinolinium.

The ion at *m*/*z* 314 was from peak 6 that eluted at a retention time of 17.9 min. The HRMS measurement of peak 6 was 314.2474 (C_21_H_32_ON, −1.404 ppm). The ion at *m*/*z* 627.4882 [2M+H]^+^ was found as well in its MS spectrum. The protonated ion produced a base peak at *m*/*z* 173 in the MS^2^ spectrum. This compound was characterized as evodiaxinine according to [14].

The ion at *m*/*z* 340 was from peak 7 that was eluted at a retention time of 18.4 min. The HRMS measurement of this peak was 340.2632 (C_23_H_34_ON, −0.865 ppm). The [2M+H]^+^ ion of the compound at *m*/*z* 679.5199 was also observed in the MS spectrum. In the MS^2^ spectrum, the protonated ion at *m*/*z* 340 yielded a base peak at *m*/*z* 186. This peak was characterized as evocarpine [14].

The ion at *m*/*z* 366 was from peak 8, occurring at a retention time of 18.8 min. HRMS measurement of the peak gave a [M+H]^+^ ion at *m*/*z* 366.2788 (C_25_H_36_ON, −0.932 ppm). The protonated ion yielded a base peak at *m*/*z* 186 in the MS^2^ experiment. According to [14], this compound was characterized as 1-methyl-2-pentadecadienyl-4(1*H*)-quinolone.

## 3. Experimental Section

### 3.1. Samples and Reagents

Twenty four batches of EF samples were collected from different regions in China (Table 1). All the samples were dried in shade and botanically authenticated. Acetonitrile (Tedia, Fairfield, OH, USA), methanol (Tedia), and formic acid (Roe Scientific Inc, Waltham, MA, USA) were MS grade. Water was Robust pure water (Le Bai Shi Food and Beverage Ltd., Guangzhou, China). All other regents were of analytical grade.

### 3.2. Sample Preparation

One hundred milligrams of each sample were accurately weighed and extracted using methanol-water (5.0 mL, 50/50, *v*/*v*) in a 15 mL-centrifuge tube for 60 min at room temperature with sonication (KQ5200E, 40 kHz, 200 W, Kunshan Ultrasonic Instrument Com. Ltd., Kunshan, China). The slurry mixture was centrifuged (TGL-16M high rate low temperature centrifuge, Changsha Maijiasen Instrumental and Equipment Com. Ltd., Changsha, China) at 10,000 rpm for 15 min. The supernatant was diluted for 100 times and filtered through a 0.45 µm PVDF syringe filter for analysis.

### 3.3. Apparatus and Parameters

#### 3.3.1. FIMS

The FIMS system consisted of a TSQ Quantum Ultra triple stage quadrupole mass spectrometer (Thermo Fisher Scientific Inc., Waltham, MA, USA) with an Accela 1250 UHPLC system equipped with an Accela 1250 photo diode array (PDA) detector, an Accela HTC PAL auto-sampler, and an Accela 1250 binary pump. No analytical column was used for this method. Diluted sample extracts were directly injected into the MS using LC auto-sampler. A C_18_ reversed-phase guard column was used primarily as an online filter to minimize the potential contamination for the MS system. The FIMS fingerprint for each sample was obtained as the average of all the spectra of a sample analyzed in 3.0 min. Mobile phase consisting of acetonitrile containing 0.1% formic acid (A) and water containing 0.1% formic acid (B) with isocratic elution at 50:50 (*v*/*v*) was used. The flow rate was 0.2 mL/min. The injection volume was 5 µL. The MS instrument parameters were as follows: sheath gas flow rate, 40 (arbitrary units); auxiliary gas flow rate, 20 (arbitrary units); spray voltage, 3000 V; capillary temperature, 300 °C; capillary voltage: 35 V; tube lens offset: 110 V. The ESI was performed in positive mode from *m*/*z* 100 to 1000 to obtain the MS fingerprints.

#### 3.3.2. Ultra High-Performance Liquid Chromatography High-Resolution Mass Spectrometry

The UHPLC-HRMS system consisted of a Thermo LTQ Orbitrap XL mass spectrometer with an Accela 1250 binary pump and a PAL-HTC-Accela auto-sampler. The chromatographic separation was carried on a Thermo Hypersil GOLD^TM^ aQ analytical HPLC column (200 × 2.1 mm, 1.9 μm) with a flow rate of 0.3 mL/min. Mobile phase A consisted of 5 mmol ammonium acetate in water and B consisted of acetonitrile. The elution gradient was 10% B (*v*/*v*) over 0–1 min and 10% to 95% B over 1–20 min. ESI was performed in positive ion mode to obtain the HRMS data using fourier transform MS (FTMS). The conditions for FTMS were as follows: sheath gas flow rate, 50 AU; aux and sweep gas, 5 AU; spray voltage, 3.0 kV; capillary temperature, 300 °C. The mass range was from *m*/*z* 100 to 1000 with a resolution of 30,000 and maximum ion injection time of 500 ms. The most intense ions (the 1st and the 2nd most intense ions) were selected for the data-dependent scan with collision energy at 35%.

### 3.4. Data Acquisition

Mass spectrometric fingerprints were obtained in positive mode. Each spectrum was averaged in a 3.0 min interval (0~3.0 min) that eluted the total ion current (TIC). Three repeat analyses of the twenty four different samples provided 72 spectra.

### 3.5. Data Processing

The mass spectrometric fingerprint of each sample was a vector (ion counts with respect to mass-to-charge ratio for a range from *m*/*z* 100 to 1000). They were exported to Excel (Microsoft, Inc., Belleview, WA, USA) for preprocessing and then to SOLO (Eigenvector Research, Inc., Wenatchee, WA, USA) for principal component analysis (PCA). The processes in Microsoft Excel involved combining the 72 spectra, sorting the data by sample names, and aligning the masses.

## 4. Conclusions

Study on geo-herbalism of an herb is one of the most popular topics in Chinese medicine. Generally, a geo-authentic herb is supposed to be of good quality, high clinical curative effect, and is traditionally recognized as a characterized regional herb. However, the assessment of geo-authentic herbs usually depends on morphological identification by a pharmacist, which needs years of experience. Moreover, the evaluation criteria of the geo-herbalism of an herb are of various kinds, e.g., quality, potency and yield. The above two points lead to big difficulties for common scientists in evaluating the geo-herbalism of an herb. Based on this issue, we think homogeneity is one of the good indicators to evaluate the geo-herbalism of an herb. EF is a very common herbal medicine and recognized as a geo-authentic herb of Guizhou Province. However, no study has been found to verify the geo-herbalism of EF. FIMS combined with chemometrics was used in the present study for grouping the EF samples from different regions rapidly and efficiently. We can easily see the differences between EF samples in a PCA scores plot. With the aid of loadings plots and HRMS data, the characteristic chemical compounds were identified. The results will provide a good basis for geo-authentication if combined with the herb’s bioactivities.
